# Chimpanzees follow human pointing when attention is engaged

**DOI:** 10.1016/j.isci.2026.117195

**Published:** 2026-08-19

**Authors:** Alicia P. Melis, Federico Rossano, Amanda Goguen, Ishmael Ayebazibwe

**Affiliations:** 1 Department of Experimental Psychology, University College London, London, UK; 2 Department of Cognitive Science, University of California, San Diego, San Diego, CA, USA; 3 Psychological and Brain Sciences, University of California, Santa Barbara, Santa Barbara, CA, USA; 4 Chimpanzees Sanctuary Welfare Conservation Trust, Entebbe, Uganda

**Keywords:** pointing, referential communication, chimpanzees, attention, object-choice tasks

## Abstract

Pointing is central to human communication and cooperative information sharing. By 12–14 months, infants understand adults’ helpful communicative intentions when they point to the location of a hidden toy. In contrast, decades of ape pointing studies have yielded negative results, leading to the influential claim that chimpanzees do not understand cooperative communicative intentions. We test an alternative account proposing that limitations in attentional engagement, rather than socio-cognitive competence, constrain chimpanzees’ performance in pointing tasks. Chimpanzees (*N* = 9) successfully used cooperative pointing to locate hidden food when ostensive name-calling and species-typical food vocalizations accompanied the gesture but failed in a closely matched condition lacking attention-grabbing auditory cues. These results are in line with the hypothesis that chimpanzees understand cooperative communicative intentions and suggest that the field may have misdiagnosed the source of prior failures, as insufficient attentional engagement, rather than absent socio-cognitive competence, may be the key limiting factor.

## Highlights

• Chimpanzees used human pointing to find hidden food with attention-grabbing cues
• Success required name-calling, food calls and pointing together
• Group success (73%) matched human infants aged 14–18 months
• Attentional engagement, not enculturation, explains success or failure

## Graphical abstract

**Figure:**
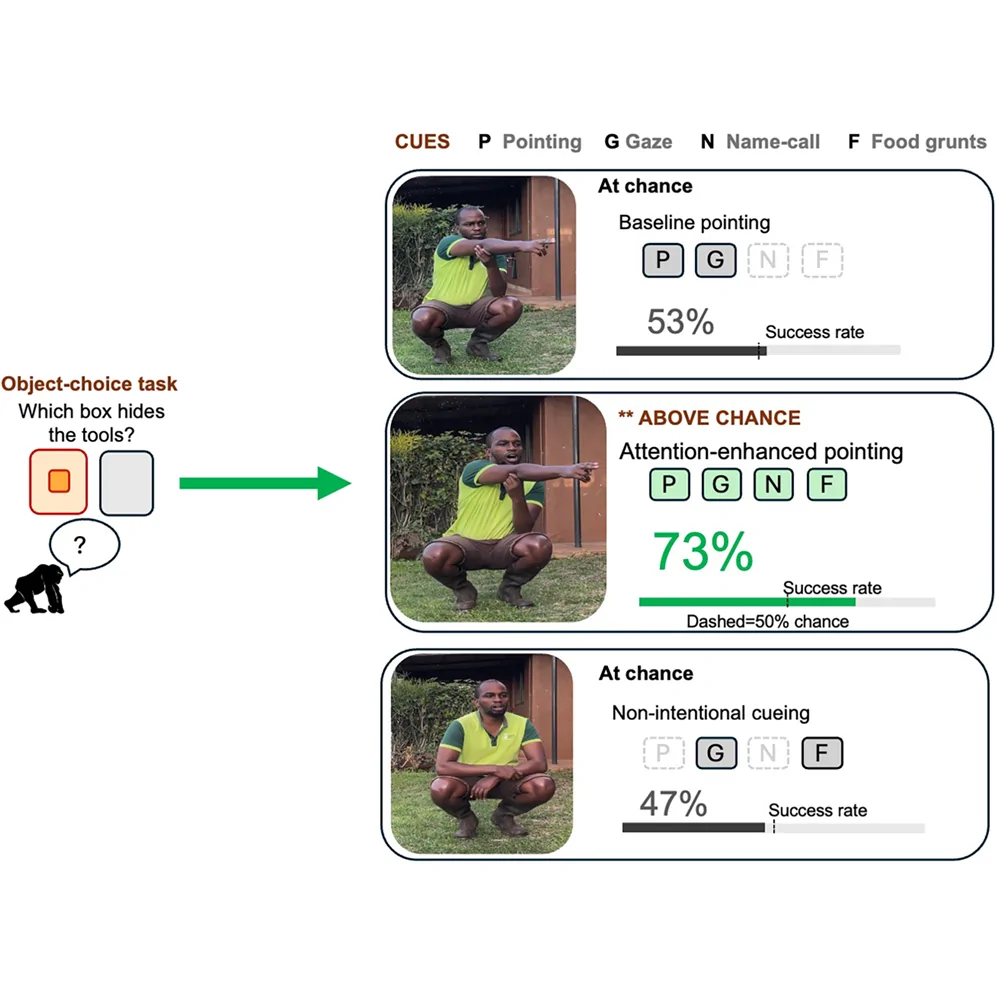

## Teaser

Behavioral neuroscience; Cognitive neuroscience

## Introduction

Pointing comprehension and production are central to human communication and cooperative information sharing. By 12–14 months of age, infants understand adults’ helpful communicative intentions when they point to the location of a hidden toy.^1^ In contrast, one of humans’ closest primate relatives, the chimpanzee, has consistently failed at pointing comprehension, with only rare exceptions reported in the literature.^2^^,^^3^^,^^4^ The *cooperative communicative intentions deficit hypothesis* proposes that chimpanzees’ failures in pointing comprehension reflect a fundamental limitation in their ability to infer others’ cooperative communicative intentions.^5^^,^^6^^,^^7^^,^^8^ Although chimpanzees readily follow gaze and monitor others’ attentional states,^9^ they are thought to lack the capacity to recognize that a human experimenter’s pointing gestures are intended to be informative and helpful rather than merely behavioral movements.^6^^,^^10^^,^^11^ In humans, recognition of communicative intentions requires sensitivity to ostensive signals—signals that explicitly mark an action as communicatively relevant, directed at a recipient, and intended to convey information. For a signal to count as communicative, the signaler must not only intend to elicit a response but must also intend for the recipient to recognize that intention.^12^^,^^13^ Ostensive cues such as eye contact, infant-directed speech, or name-calling, play a central role in human communication and in the natural pedagogy system that enables infants to learn from caregivers.^14^^,^^15^ In contrast, it has been argued that nonhuman primates show little evidence of either producing or understanding ostensive signals.^5^

Given the central role of ostensive engagement in human communication, an alternative explanation that has remained largely theoretically underdeveloped and has never been systematically tested is that chimpanzees’ failures may primarily reflect limitations in attentional engagement rather than a lack of socio-cognitive competence. We term this account the *attentional deficit hypothesis*, which predicts that chimpanzees should succeed at pointing comprehension once their attention is reliably engaged and the communicative context is made sufficiently salient. To our knowledge, this prediction has never been directly tested: prior studies have neither systematically manipulated attentional engagement nor included the control conditions necessary to isolate its contribution to pointing comprehension.

Critically, the primary empirical basis for this proposed species difference comes from great apes’ poor performance in the object-choice paradigm. In this task, an experimenter provides an informative cue, such as pointing, toward one of two opaque containers to indicate the location of a hidden reward. Human infants reliably perform above chance,^16^^,^^17^ and domestic dogs generally outperform great apes and monkeys—a pattern interpreted as domestication-driven sensitivity to human ostensive and communicative cues,^18^ though this interpretation has been challenged on methodological grounds.^19^ However, great apes consistently perform at chance levels,^11^^,^^20^^,^^21^ and while some positive results have been reported,^4^^,^^22^ these remain inconsistent across studies. With the exception of the enculturation hypothesis,^23^ no systematic theoretical account has been proposed to explain what drives success in those cases, leaving open the question of whether factors such as repeated cue exposure or stimulus enhancement, where the pointing finger is positioned directly adjacent to the baited option, may have contributed to positive findings.^4^^,^^24^^,^^25^^,^^26^

We propose that the patchy pattern of positive findings is not random. Rather, across studies, success disproportionately occurs under conditions that facilitate attentional engagement, a factor that has not been systematically identified as a unifying explanation. Great apes with extensive histories of interaction with humans outperform non-enculturated apes.^4^^,^^23^^,^^27^^,^^28^ Although this pattern has been interpreted as evidence that, as in human children and dogs, extensive interactions with humans are necessary for apes to acquire pointing comprehension skills,^19^^,^^23^^,^^24^ we offer a different interpretation. We suggest that enculturation may increase both apes’ motivation to attend to humans and their sensitivity to cooperative intentions. Chimpanzees more readily follow pointing-like gestures in competitive than in cooperative contexts,^10^^,^^11^ a pattern consistent with heightened attentional monitoring of social partners when they are potential competitors. There is some evidence that spatial set-ups using distal or peripheral configurations may increase performance relative to proximal arrangement.^29^^,^^30^ Proximal arrangements typically used in ape studies may interfere with subjects’ ability to attend to the experimenter’s cues, whereas distal configurations, which are standard in dog studies,^27^ are more likely to support cue detection and tracking. Performance also improves when pointing gestures are continuous rather than momentary,^27^^,^^29^ likely because prolonged cues are more readily detected and tracked. Beyond spatial configuration, auditory cues facilitate success when paired with indexical cues such as gaze or pointing.^25^^,^^31^ Together, these findings suggest that conditions enhancing attentional engagement may increase the likelihood that chimpanzees will detect, track, and use humans’ informative gestures.

Building on this interpretation, we hypothesize that although chimpanzees are sensitive to others’ gaze and attentional states, in food-search contexts, they normally adopt a “foraging mindset” that deprioritizes attending to others unless they are potential competitors. Under such conditions, an experimenter’s actions may simply fall outside their attentional priorities. The *attentional deficit hypothesis*, therefore, predicts that when attention is sufficiently engaged, through salient, ostensive, and motivationally relevant cues, chimpanzees should be more likely to infer that the human is attempting to communicate cooperatively.

In the present study, we tested this prediction using a pointing context designed to make the human’s cooperative communicative gestures maximally salient and perceptible. We combined pointing with two attention-grabbing cues: ostensive name-calling and food grunts. We compared this condition with two controls: (1) a baseline pointing condition without vocal cues and (2) a non-intentional cueing condition that only included gaze and food grunts. Chimpanzees were tested in a distal set-up using continuous contralateral pointing, in which the experimenter’s arm crossed the body midline toward the target location, reducing the spatial asymmetry between the two options and thereby minimizing the possibility of local enhancement. We predicted superior performance in the attention-enhanced condition relative to both control conditions. The results show successful performance only in the attention-enhanced condition, suggesting that chimpanzees understand cooperative communicative intentions once their attention is fully engaged.

## Results

**Figure 1:**
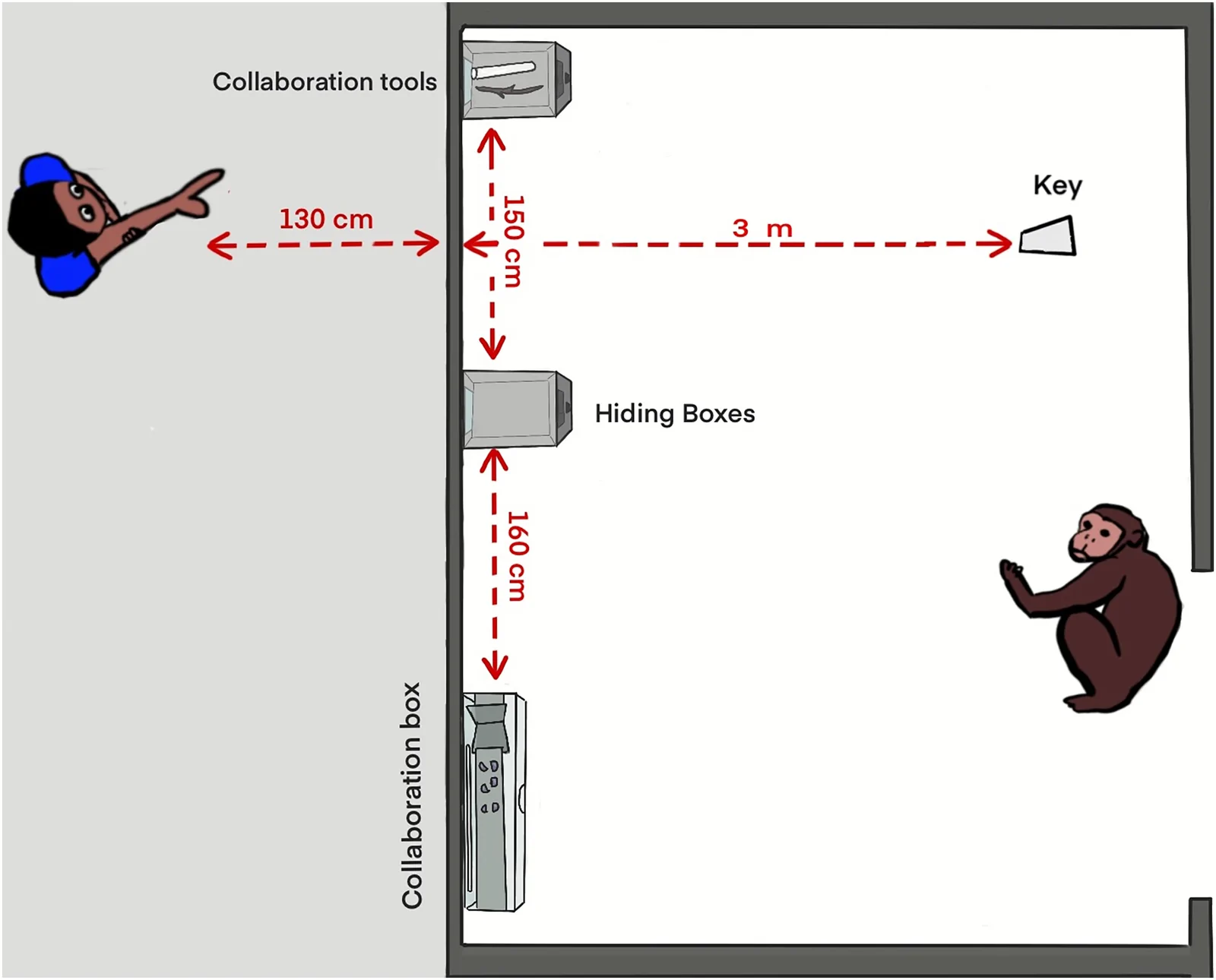
Test setup Diagram of the test setup showing the location of the apparatuses, the key, the positions of the subject, and experimenter 1 (E1) during the test phase. One of the hiding boxes (150 cm apart) contained the two tools necessary to obtain the food rewards inside the collaboration box. Subjects needed to use the key placed on the ground to open the hiding box, extract the tools, and then use them at the collaboration box together with the human experimenter to obtain the rewards. After entering the room, subjects retrieved the centered key and chose one hiding box. Incorrect choices ended the trial, whereas correct choices led to collaboration with E2 to get the grapes. Each subject completed two sessions per condition (one session/day), each consisting of six test trials (12 trials per condition, 36 total), with condition order counterbalanced across individuals according to a Latin square design and one session conducted per day.

**Figure 2:**
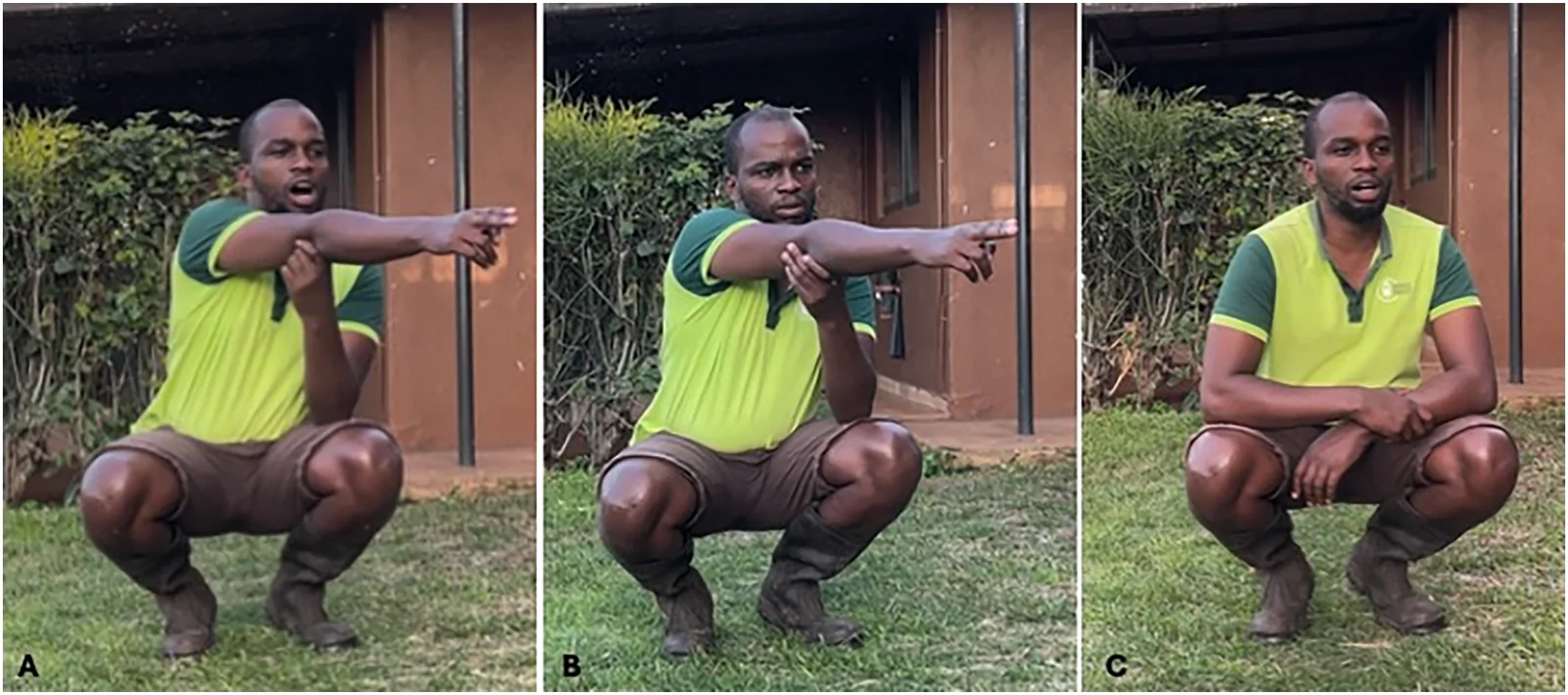
Experimenter’s cues across conditions Pictures of the three conditions. (A) Attention-enhanced pointing with name-calling and food grunts. (B) Baseline pointing (without name-calling and food grunts). (C) Non-intentional cueing, with gaze and food grunts (without name calling and pointing).

Nine chimpanzees were presented with two identical hiding boxes and a collaboration box affixed to the bars of the testing room. The tools required to obtain the food rewards from the collaboration box were hidden in one of the hiding boxes, and subjects were provided a key that would allow them to open one of the two boxes. This key, once inserted into a box, could not be removed by the subject to open the second box (Figure 1). The experimenter squatted at an equal distance from the boxes and provided specific communicative signals, depending on the condition (Figure 2). In the attention-enhanced pointing, the experimenter called the subject’s name, gazed at the correct box, and produced “food-grunt” noises, in addition to the continuous contralateral pointing and slight body orientation. In the baseline pointing, the experimenter provided no audible cues, and they engaged only in slight body orientation, continuous contralateral pointing, and gazing. In the non-intentional cueing, the experimenter only gazed and produced “food-grunts” (no pointing and no name-calling).

### Group-level performance across conditions

**Figure 3:**
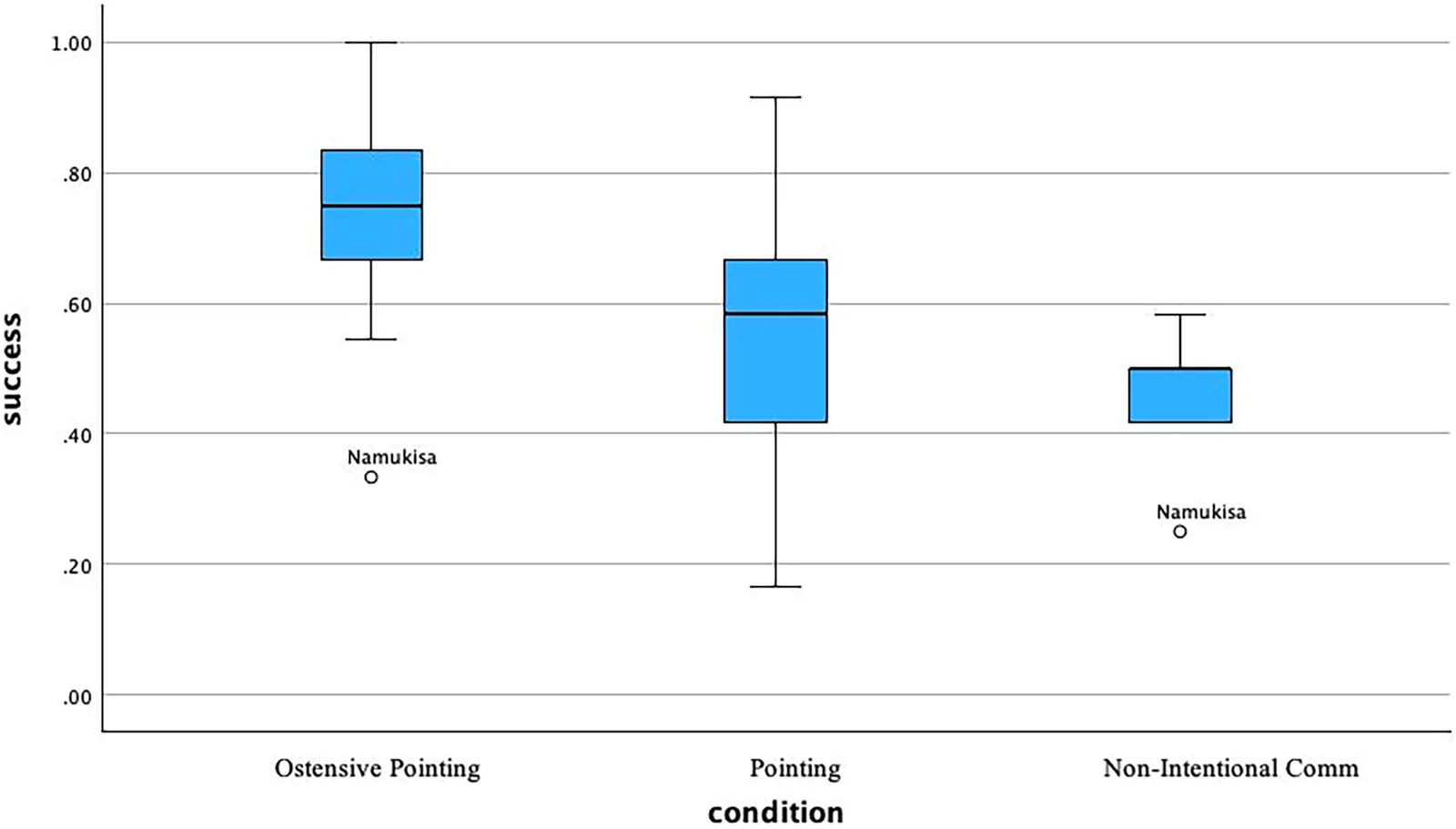
Success rates across conditions Boxplot showing success rates across conditions, *N* = 9. The center line represents the median, box edges represent the 25th and 75th percentiles (interquartile range), and whiskers extend to the largest and smallest values within 1.5 times the interquartile range. Circles represent the outlier.

Subjects were successful on average in 73% of trials in the attention-enhanced pointing condition, 53% in the baseline pointing condition, and 46% in the non-intentional cueing condition (Figure 3). A comparison against chance (50%) showed that performance was significantly above chance only in the attention-enhanced pointing condition (Wilcoxon signed-rank test: Z = −2.38, *p* = 0.016).

A generalized linear mixed-effects model with a binomial logit link was fitted to examine the effects of Condition, condition order, trial number, and baited-box side on accuracy, with a random intercept for Subject. Including Condition significantly improved model fit relative to a model without this predictor, χ^2^(2) = 19.17, *p* < 0.001, indicating that accuracy significantly differed across conditions. Relative to the attention-enhanced pointing condition, subjects were 62% less likely to respond correctly in the baseline pointing condition (OR [odds ratio] = 0.38, 95% confidence interval [CI] [0.21, 0.69], *p* = 0.002) and 71% in the non-intentional cueing condition (OR = 0.29, 95% CI [0.16, 0.52], *p* < 0.001). None of the control variables significantly predicted accuracy (all *s*s > 0.28). A nonparametric Friedman test yielded the same pattern of results, confirming the robustness of the effect.

### Individual differences in performance

Individual-level binomial tests (two-tailed, *n* = 12, chance = 0.50) showed that in the Attention-enhanced Pointing condition, three individuals performed above chance: Indi (11/12; *p* = 0.006), Medina (12/12; *p* < 0.001), and Pasa (10/12; *p* = 0.038). In the baseline pointing condition only one subject (Indi) showed above-chance performance (11/12; *p* = 0.006). In the non-intentional cueing condition, no subject reached above-chance levels (all scores ≤7/12; *p* ≥ 0.39). Although only a few individuals achieved statistically significant performance on their own, most subjects showed numerically higher scores in the Attention-enhanced Pointing condition compared to the other conditions: six of nine subjects scored nine or more correct choices in the attention-enhanced condition, compared to one of nine in the baseline pointing condition and none in the non-intentional cueing condition (Table 1).

**Table 1:** Individual performance showing the number of correct trials (maximum = 12) in each condition

Subject	Attention-enhanced pointing	Baseline pointing	Non-intentional cueing
Asega#	9∗∗	8	7
Baluku#	9∗∗	8	5
Becky	8	7	6
Indi#	11∗	11∗	6
Kidogo#	6	3	5
Medina	12∗	7	6
Namukisa	4	2	3
Nkumwa	9	6	5
Pasa	10∗	5	7

∗Individuals performing significantly above chance based on two-tailed binomial tests (*p* < 0.05). ∗∗Individuals reaching significance under one-tailed binomial tests (*p* < 0.05) Based on our directional prediction of above-chance performance in the attention-enhanced pointing condition, a score of 9/12 or above reaches significance under one-tailed binomial tests (*p* = 0.037), yielding six out of nine subjects performing above chance in that condition, compared to one in the baseline pointing condition and none in the non-intentional cueing condition. #Individuals that participated in Kirchofer et al.^20^

## Discussion

Subjects performed significantly above chance only in the attention-enhanced pointing condition, which differed from the baseline pointing condition solely by the addition of attention-grabbing auditory cues, namely ostensive name-calling and food grunts. A similar combination of cues has also led to successful performance in another study,^31^ although that study did not include control conditions to isolate the effect of attentional engagement. Together, these results indicate that successful performance depends on the combination of an indexical cue, i.e., pointing, with ostensive, auditory attention-enhancing cues. The indexical signal alone is not sufficient. Rather, chimpanzees’ attention must first be engaged and directed to the communicative act (e.g., via name-calling), after which the pointing gesture can specify the intended referent within a meaningful communicative context (e.g., food-associated vocalizations). In combination, these cues support both attentional engagement and referential interpretation, thereby enhancing performance. Critically, the same individuals who succeeded in the Attention-enhanced condition performed at chance in the non-intentional condition, which included food grunts and gaze cues but lacked the indexical gesture. This pattern indicates that, in the absence of explicit ostensive and indexical signals, chimpanzees do not reliably identify the intended referent. In other words, attention-grabbing cues alone are insufficient: successful comprehension requires both a salient indexical gesture and sufficient attentional engagement to detect and interpret the signal. Future studies could isolate the individual components of the attention-enhanced cue, for instance by testing subsets of cues rather than the full combination. However, given that previous studies using name-calling alongside pointing have not consistently yielded positive results,^11^ we suspect it is the cumulative effect of multiple attention-grabbing cues that is key.

All three conditions employed the same distal spatial set-up, yet subjects succeeded only in the Attention-enhanced condition. This pattern indicates that although distal arrangements can facilitate performance,^21^^,^^30^^,^^32^ they are not sufficient on their own. Additional factors that draw subjects’ attention to the experimenter therefore appear to be necessary for success. In the present study, attention was enhanced through name-calling and food-associated vocalizations. Prior work using event-related potentials has shown that hearing one’s name elicits an immediate attention response in chimpanzees.^33^ Moreover, eye-tracking studies have demonstrated that both ostensive and non-ostensive attention-getters facilitate gaze following to a target object at comparable levels, while ostensive cues (such as name-calling) also increase overall attention to objects in the environment.^34^ Our study extends this work by showing that when such attentional engagement is combined with a clear indexical communicative cue (i.e., pointing) chimpanzees can identify the intended referent. Food-associated vocalizations may further increase motivational relevance or attentional engagement, but the present data do not allow these functions to be dissociated.

The chimpanzees in our study were sanctuary-housed and, following Clark and colleagues,^27^ only “occasionally” exposed to humans rather than truly enculturated. They therefore differ from language-trained or fully enculturated chimpanzees, who have typically been described as those most capable of understanding human pointing cues.^4^^,^^23^ The traditional enculturation argument holds that extensive immersion in human environments, such as those of human children or pet dogs, is necessary for developing comprehension of human communicative gestures.^19^ However, our findings suggest that strong enculturation is not required. These results show that once subjects’ attention is successfully engaged, chimpanzees can use humans’ informative gestures. This raises the possibility that the difference between enculturated and non-enculturated chimpanzees may lie less in their long-term opportunity to learn associations between pointing and rewards,^24^^,^^28^ and more in how readily they attend to human partners. Individual differences in their relationships with, and attention to, humans may be the critical factor driving performance on these tasks.

Consistent with this interpretation, Hopkins et al.^22^ found no effect of rearing history on object-choice task performance in a large sample of chimpanzees (*N* = 138) and reported that both the combined cues condition (pointing with food grunts and name calling) and the pointing-alone condition (pointing with name-calling) outperformed the vocal-alone condition (food grunts without pointing), a pattern broadly consistent with our findings in that an indexical gesture appears necessary for success. However, success in Hopkins et al.^22^ is open to at least two alternative explanations. First, their procedure used proximal ipsilateral pointing, with the index finger placed immediately next to the target, meaning stimulus enhancement cannot be ruled out. Second, most of their subjects had participated in numerous prior cognitive studies, and the authors acknowledge that this testing history may have contributed to individual variation in performance. The study found a significant group-level effect, with 43% of individuals passing, though this figure was calculated across all three conditions combined, leaving it unknown how subjects performed under each condition separately. This is particularly relevant because under our attentional engagement account, success in the pointing-alone condition would be unexpected in naive subjects. Beyond these observations, Hopkins et al.^22^ do not propose a specific account that would explain the full pattern of results. Our attentional engagement account goes further in providing a specific explanation: success depends on whether the communicative context is sufficiently salient to secure the subject’s attention. We suggest that prior testing experience in the Hopkins et al. sample may have implicitly fostered attentional responsiveness to human communicative cues, effectively substituting for the explicit attention-grabbing cues that were necessary to achieve success in our naive sanctuary chimpanzees. The use of distal contralateral pointing in our study further rules out stimulus enhancement as an alternative explanation, providing stronger evidence for referential comprehension of pointing.

The results suggest chimpanzees can use human communicative gestures to find hidden rewards when the signals are sufficiently explicit, both in attracting attention to the communicator and in providing clear indexical cues such as pointing and gaze. This level of success at the group level (73%) is comparable to performance reported in young children in similar paradigms, falling between that observed at 14 and 18 months of age (64% and 77%, respectively).^16^ This pattern shows that the main limitation in chimpanzees’ performance in object-choice tasks is not a failure to understand communicative intentions per se, but rather a minimal motivation to attend to the communicator’s signals, particularly when they are in a “foraging mindset”.

A closely analogous pattern has been shown in young human children. Kachel and colleagues^35^ showed that two-year-old children reliably attend to pointing gestures produced by adults but perform at chance when the same gestures are produced by a two-year-old peer. Together, these findings suggest that social expectations regarding who is producing the signal, such as trust, relationship, and perceived relevance, play a crucial role not only for young human children but also for chimpanzees. Consistent with this, there is also evidence that chimpanzees and human infants show reduced motivation to follow allospecific gaze compared to bonobos, orangutans, and human adults who follow conspecific and allospecific gaze at similar levels,^36^ further suggesting that perceived social relevance shapes whose signals are attended to. When human communicators provide cues that capture subjects’ attention and establish the signal’s relevance to the recipient, chimpanzees can use human pointing gestures to solve object-choice tasks. Finally, by demonstrating that communicative success depends on the combination of visual and vocal cues, our findings underscore the importance of embodied and multimodal communication. In natural interactions, communicative signals rarely occur in isolation: gestures, gaze, vocalizations, and contextual cues work together to capture attention, indicate intent, and disambiguate meaning.^37^^,^^38^ Our results suggest that chimpanzees also rely on this multimodal integration to interpret human referential acts effectively.

### Limitations of the study

Although it is possible that the attention-enhancing cues acted cumulatively, the current study does not allow exactly pinpointing the individual attentional impact of name-calling and food grunts. Future studies could try to decompose their role by running parallel conditions with just one auditory cue at a time (e.g., pointing with food grunts only; pointing with name-calling only), which would help determine whether it is the cumulative effect of multiple attention-grabbing cues that is critical for securing sufficient attentional engagement.

## Resource availability

### Lead contact

Requests for further information and resources should be directed to and will be fulfilled by the lead contact, Alicia P. Melis (a.melis@ucl.ac.uk).

### Materials availability

This study did not generate new materials.

### Data and code availability

• All data reported in this study are available in the supplemental information.
• This study does not report original code.
• Any additional information required to reanalyze the data reported in this study is available from the lead contact upon request.

## Acknowledgments

We are very thankful to Dr. Joshua Rukundo, Titus Mukungu, and the trustees and all the staff of the Chimpanzee Sanctuary and Wildlife Conservation Trust (CSWCT) for their continuous help and support. We appreciate the hard work of the animal caregivers and their support in helping us conduct the experiments. We also thank Martin Millson for building the apparatuses and Evan Gurkin for reliability coding. We also appreciate permission from the Uganda National Council for Science and Technology (NS360) and the Uganda Wildlife Authority for allowing us to conduct our research in Uganda (Research permit UWA/COD/96/05).

## Author contributions

Conceptualization, A.P.M. and F.R., methodology, A.P.M., F.R., and A.G., investigation, A.G. and I.A., visualization, A.P.M. and A.G., funding acquisition, A.P.M., project administration, A.P.M. and A.G., supervision, A.P.M. and F.R., writing – original draft, A.P.M., F.R., and A.G., writing – review and editing, A.P.M., F.R., A.G., and I.A.

## Declaration of interests

The authors declare no competing interests.

## Declaration of generative AI and AI-assisted technologies in the writing process

During the preparation of this manuscript, the authors used Claude to improve the language, grammar, and readability of the manuscript, and to check spelling and clarity. The authors reviewed and edited the output as needed and take full responsibility for the content of the published article.

## STAR★Methods

### Experimental model and study participant details

Nine chimpanzees (six females, three males; aged 26–41 years old) from the Ngamba Island Chimpanzee Sanctuary (https://ngambaisland.org) in Uganda participated in this study. All chimpanzees at the sanctuary were either orphaned or rescued, and they come from diverse backgrounds, including the entertainment industry and the illegal pet trade. The sanctuary spans 95 acres of land where the chimpanzees can roam freely during the day. In the evening, the chimpanzees have the option to return from the forest and enter the outdoor holding facilities, where they can sleep in hammocks. The holding facilities are also used as the researchers’ testing site. When the other chimpanzees are released in the morning, study subjects stay in the holding facilities until they complete their testing for the day. Sanctuary staff feed the chimpanzees an assortment of fruits multiple times a day. Any food provided during testing is supplementary to their regular diet, and they are not restricted from eating their regular meals. All nine subjects had familiarity with the apparatuses used in this study,^39^ and four subjects were previously tested in an object-choice pointing study.^20^

**Table:** Subjects’ names, sex and age.

Subject	Sex	Age
Asega	M	26
Baluku	M	26
Becky	F	33
Indi	M	25
Kidogo	F	40
Medina	F	20
Namukisa	F	25
Nkuumwa	F	28
Pasa	F	25

### Method details

The experimental setup consisted of two identical hiding boxes and a collaboration box fixed to the bars of the testing room. Each hiding box had a transparent window facing the bars. The other side of the box had a drop-down door and a keyhole where a key could be inserted top open the door and reveal the contents of the box. Once the key was inserted in one box, it could not be removed, thus allowing only one choice per trial. During pretest and test phases, one hiding box contained a raking tool and a cylindrical pushing tool required to obtain grapes from the collaboration box. The collaboration box consisted of an upper platform holding the grapes and a lower compartment with a retrieval opening. To obtain the rewards, subjects first had to pass the raking tool to the experimenter, who pushed the grapes onto a small platform, after which subjects used the pushing tool to tilt the platform and retrieve the grapes from the lower compartment.

The pretest re-familiarized subjects with the collaboration box and the hiding-box contingencies, including that only one box contained the collaboration tools and that the box’s contents were visible from one side. The hiding boxes were affixed to the bars between the testing room and an adjacent room accessible to the chimpanzees, allowing subjects to inspect the box’s contents from behind before choosing. While a caretaker distracted the subject (i.e., engaged their attention to prevent observation of the baiting process), E1 baited one box with the tools and placed grapes in the collaboration box. Subjects obtained the key to open the hiding boxes in the adjacent room, from where they could view the box contents, returned to the testing room, and opened one box (Figure S1). If they chose the correct box, they proceeded to use the tools with E2 to obtain the grapes; if not, the trial was ended. Subjects completed one session of six trials.

During test sessions, the collaboration box remained in place, whereas the hiding boxes were moved to the same side of the testing room as the collaboration box, positioned 150 cm apart with their transparent windows facing the human corridor. The nearest hiding box was positioned 160 cm from the collaboration box. The key was placed equidistant from both hiding boxes and 3 m from the plane formed by the boxes (Figure 1).

E1 baited the collaboration box with grapes and one of the hiding boxes with the tools while a caregiver distracted the subject in a waiting room. Each session began with four warm-up trials in which no tools were hidden, and no communicative cues were provided. During warm-up trials, the subject had 30 s to retrieve the key and use it to open one of the two unbaited hiding boxes. E2 stood to the side of the testing area and provided no communicative cues. After the subject opened one of the hiding boxes, the trial ended, and the subject was called back to the waiting room. The purpose of the warm-up trials was to ensure that box opening in the absence of information was not rewarded.

In test trials, E1 baited one of the two hiding boxes, counterbalancing the baited location across trials and ensuring that the same box was not baited on two consecutive trials. After baiting, E2 squatted down 130 cm from the bars and, depending on condition, provided communicative cues before the subject entered the room to begin the trial (Figure 2).

#### Attention-enhanced pointing

The experimenter called the subject’s name, gazed at the correct box, produced four food-grunt vocalizations, and engaged in continuous contralateral pointing with slight body orientation (Videos S1 and S4).

Video S1. Re-enactment of the attention-enhanced pointing condition, front view

Video S4. Example test trial showing subject Medina in the attention-enhanced pointing condition Medina follows the communicative gestures and locates the tools, then moves to the collaboration box and, together with the experimenter, extracts the rewards.

#### Baseline pointing

The experimenter provided no audible cues and engaged only in continuous contralateral pointing, slight body orientation, and gazing (Videos S2 and S5).

Video S2. Re-enactment of the baseline pointing condition, front view

Video S5. Example test trial showing subject Baluku in the baseline pointing condition Baluku does not follow the communicative gesture and opens the empty hiding box.

#### Non-intentional cueing

The experimenter only gazed at the box with slight body orientation and produced four food-grunt vocalizations, without pointing or name-calling (Videos S3 and S6).

Video S3. Re-enactment of the non-intentional cueing condition, front view

Video S6. Example test trial showing subject Namukisa in the non-intentional cueing condition Namukisa does not follow the communicative cues and opens the empty hiding box.

Once E2 was ready to provide the communicative cues, the caregiver, who had been distracting the subject in the waiting room, opened the door to the testing room. The trial began when the subject entered the room. After entering, subjects retrieved the centrally placed key and chose one hiding box. Incorrect choices ended the trial, and the subject was returned to the waiting room while E1 prepared the next trial, whereas correct choices led to collaboration with E2 to obtain the grapes. After the subject obtained the grapes, they were returned to the waiting room while E1 prepared the subsequent trial. Each subject completed two sessions per condition, each consisting of four warm-up and six test trials (12 trials per condition; 36 trials total). Condition order was counterbalanced using a Latin square design, with one session conducted per day.

### Quantification and statistical analysis

Inter-observer Reliability: A.G and I.A coded choices live and subsequently verified the coding using the video recordings. An independent third observer, blind to the study hypotheses, coded 20% of all trials from the video files to assess inter-observer reliability. Inter-rater reliability for box choice (left vs. right) was excellent (Cohen’s κ = 0.97), with 98% agreement across 63 double-coded trials.

To examine the effects of experimental condition, order, trial number, and baited side on accuracy, we fitted a logistic generalized linear mixed-effects model (GLMM) using maximum likelihood estimation.^40^ Models were implemented in R (version 4.5.2) using the glmer function from the lme4 package.^41^ Accuracy was modeled as a binary response variable (1 = correct, 0 = incorrect) with a binomial error structure and logit link function. The full model included Condition (Attention-Enhanced Pointing, Baseline Pointing, Non-Intentional Cueing) as the main predictor, along with the control variables Order (order in which conditions were experienced), Trial (trial number), and Baited (side of the baited box: left or right). Subject identity was included as a random intercept. The model formula was:

Correct∼Condition+Order+Trial+Baited+(1|Subject)

Collinearity among fixed effects was assessed using generalized variance inflation factors (VIFs) computed with the vif function from the car package, applied to a model containing only fixed effects. No collinearity issues were detected (VIF_max_ = 1.01).

To evaluate the effect of Condition, we compared the full model to a null model containing only the control predictors (Order, Trial, Baited) and the random intercept. The full model provided a significantly better fit than the null model (likelihood ratio test: χ^2^(2) = 19.17, *p* < 0.001), indicating that Condition significantly predicted accuracy.

Model estimates are reported in the Table below. Relative to the Attention-Enhanced Pointing condition (reference level or intercept), participants were significantly less likely to respond correctly in the Baseline Pointing condition (β = −0.96, SE = 0.30, *z* = −3.17, *p* = 0.002), corresponding to an odds ratio (OR) of 0.38 (95% CI [0.21, 0.69]). Participants were also significantly less likely to respond correctly in the Non-Intentional Cueing condition (β = −1.24, SE = 0.30, *z* = −4.09, *p* < 0.001), with an OR of 0.29 (95% CI [0.16, 0.52]).

None of the control variables significantly predicted accuracy: Order (β = −0.05, SE = 0.15, *p* = 0.76, OR = 0.95, 95% CI [0.71, 1.28]), Trial (β = −0.03, SE = 0.04, *p* = 0.32, OR = 0.97, 95% CI [0.91, 1.04]), or Baited side (β = −0.26, SE = 0.24, *p* = 0.28, OR = 0.77, 95% CI [0.48, 1.23]).

#### Table model estimates

Fixed-effect estimates from the full generalized linear mixed-effects model predicting accuracy. Estimates, standard errors (SEs), 95% confidence intervals (CIs), and likelihood ratio tests are shown. Condition was dummy-coded with *Attention-Enhanced Pointing* as the reference level.

**Table:** 

Predictor	Estimate (β)	SE	Z	p	Odds Ratio	95% CI (OR)
Intercept	1.53	0.49	3.10	0.002	4.61	[1.76, 12.10]
Baseline Pointing	−0.96	0.30	−3.17	0.002	0.38	[0.21, 0.69]
Non-Intentional Cueing	−1.24	0.30	−4.09	<0.001	0.29	[0.16, 0.52]
Order	−0.05	0.15	−0.31	0.76	0.95	[0.71, 1.28]
Trial	−0.03	0.04	−1.00	0.32	0.97	[0.91, 1.04]
Baited (R)	−0.26	0.24	−1.09	0.28	0.77	[0.48, 1.23]

As a complementary non-parametric approach, a Friedman test was used to assess overall differences in success across the three conditions, and Wilcoxon signed-rank tests were used to compare performance in each condition against chance level (50%). At the individual level, two-tailed and one-tailed binomial tests (chance = 0.50) were conducted for each subject in each condition to determine whether individual performance differed significantly from chance.

## Supplemental information

Document S1. Figure S1

Data S1. Trial-by-trial data indicating subject, condition, order of conditions, continuous trial number across the full experiment (1–36), trial number per condition (1–12), hiding box baited (left vs. right from the subject’s perspective), side choice (left vs. right from the subject’s perspective), and choice accuracy (correct = 1, incorrect = 0)

Data S2. Proportion of successful trials per subject and condition This file contains the tabulated data behind the figures.
