# Supplementary material for: Chimpanzees follow human pointing when attention is engaged
Source: iScience. 2026 Aug 19;29(9):117195. doi: 10.1016/j.isci.2026.117195 (PMC13521074; doi:10.1016/j.isci.2026.117195)
Supplement: Supplementary file 1 [file mmc1.pdf]

iScience, Volume *29*

Supplemental information

Chimpanzees follow human pointing when attention is engaged

Alicia P. Melis, Federico Rossano, Amanda Goguen, and Ishmael Ayebazibwe

PRETEST FAMILIARIZATION

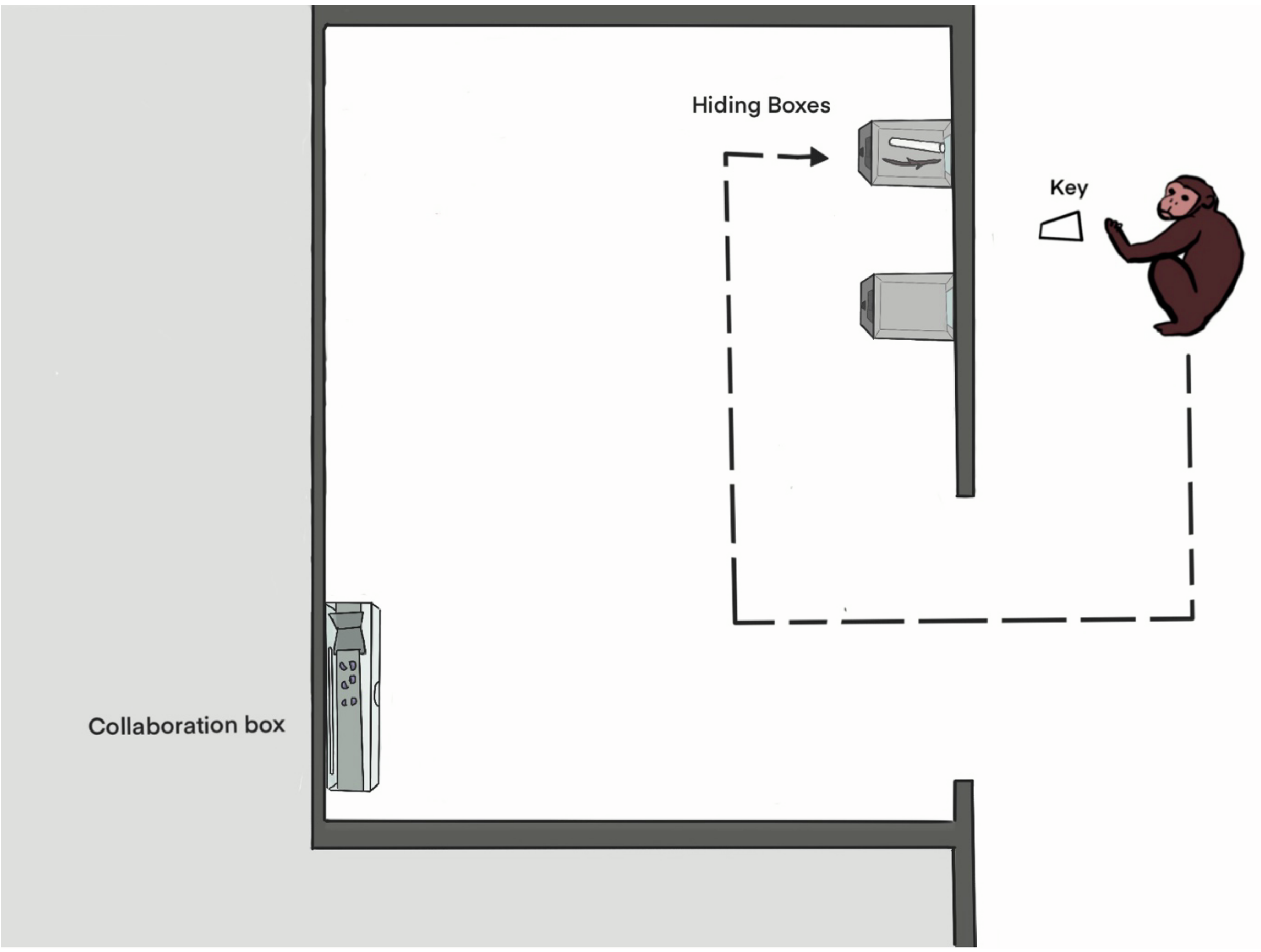

Figure S1. Diagram of the pretest familiarization setup showing the location of the apparatuses and the key used to open the hiding boxes. Subjects picked up the key placed on the ground in the room facing the back of the boxes; from this position, they could see the location of the tools. They then walked to the testing room, selected the box containing the tools, and extracted the tools required to obtain the rewards from the collaboration box. Together with the Experimenter they extracted the rewards from the collaboration box.
